# Evaluation of the PaO2/FiO2 ratio after cardiac surgery as a predictor of outcome during hospital stay

**DOI:** 10.1186/1471-2253-14-83

**Published:** 2014-09-26

**Authors:** Francisco Esteve, Juan C Lopez-Delgado, Casimiro Javierre, Konstantina Skaltsa, Maria LL Carrio, David Rodríguez-Castro, Herminia Torrado, Elisabet Farrero, Antonio Diaz-Prieto, Josep LL Ventura, Rafael Mañez

**Affiliations:** 1 Intensive Care Department, Hospital Universitari de Bellvitge, IDIBELL (Institut d’Investigació Biomèdica Bellvitge; Biomedical Investigation Institute of Bellvitge), C/Feixa Llarga s/n. 08907, Barcelona, L’Hospitalet de Llobregat, Spain; 2 Physiological Sciences II Department, Universitat de Barcelona, IDIBELL, Barcelona, Spain; 3 Department of Public Health, Universitat de Barcelona, Barcelona, Spain

**Keywords:** Cardiac surgery, Cardiopulmonary bypass, PaO2/FIO2 ratio, Outcomes, Long-term survival

## Abstract

**Background:**

The arterial partial pressure of O2 and the fraction of inspired oxygen (PaO2/FiO2) ratio is widely used in ICUs as an indicator of oxygenation status. Although cardiac surgery and ICU scores can predict mortality, during the first hours after cardiac surgery few instruments are available to assess outcome. The aim of this study was to evaluate the usefulness of PaO2/FIO2 ratio to predict mortality in patients immediately after cardiac surgery.

**Methods:**

We prospectively studied 2725 consecutive cardiac surgery patients between 2004 and 2009. PaO2/FiO2 ratio was measured on admission and at 3 h, 6 h, 12 h and 24 h after ICU admission, together with clinical data and outcomes.

**Results:**

All PaO2/FIO2 ratio measurements differed between survivors and non-survivors (*p <* 0.001). The PaO2/FIO2 at 3 h after ICU admission was the best predictor of mortality based on area under the curve (*p <* 0.001) and the optimum threshold estimation gave an optimal cut-off of 222 (95% Confidence interval (CI): 202–242), yielding three groups of patients: Group 1, with PaO2/FIO2 > 242; Group 2, with PaO2/FIO2 from 202 to 242; and Group 3, with PaO2/FIO2 < 202. Group 3 showed higher in-ICU mortality and ICU length of stay and Groups 2 and 3 also showed higher respiratory complication rates. The presence of a PaO2/FIO2 ratio < 202 at 3 h after admission was shown to be a predictor of in-ICU mortality (OR:1.364; 95% CI:1.212-1.625, *p <* 0.001) and of worse long-term survival (88.8% vs. 95.8%; Log rank *p* = 0.002. Adjusted Hazard ratio: 1.48; 95% CI:1.293–1.786; *p* = 0.004).

**Conclusions:**

A simple determination of PaO2/FIO2 at 3 h after ICU admission may be useful to identify patients at risk immediately after cardiac surgery.

## Background

In critically ill patients the PaO2/FIO2 ratio is an indicator of oxygenation status and is one of the diagnostic criteria for acute respiratory distress syndrome in adults (ARDS) [1-4]. A low PaO2/FiO2 value has been associated with increased mortality and hospital stay in patients admitted to the intensive care unit (ICU) [5-8]. The PaO2/FiO2 ratio is widely used in ICUs because it quickly and easily provides data on the oxygenation status of critically ill patients. Its values are included in ICU prognostic scores [9,10]. During surgery, atelectasis may cause intraoperative gas exchange abnormalities, which may be increased by inflammation triggered by the surgery itself, leading to postoperative lung dysfunction even in patients without preexisting lung injury. Despite protective mechanical ventilation during and after surgery, including recruitment maneuvers, a lower PaO2/FiO2 may be a reflection of a persistent lung dysfunction which can influence outcome [11].

The population of patients who undergo cardiac surgery is heterogeneous. With the trend towards greater longevity, patients are likely to be older and to present higher rates of comorbidities such as chronic heart or respiratory failure [12]. The cardiac surgery scores habitually used to predict short-term postoperative mortality (Parsonnet, European System for Cardiac Operative Risk Evaluation (EuroSCORE)) are objective and relatively simple [13,14]; however, they evaluate preoperative status and may become inaccurate in the case of surgical complications. ICU scores (Acute Physiology and Chronic Health Evaluation (APACHE) II and III, Simplified Acute Physiology Score (SAPS) II and III) [9,10] are not specific for cardiac surgery and both require 24 hours for calculation. Other than their clinical judgment, physicians have few tools available for assessing outcome immediately after cardiac surgery.

The aim of this study was to evaluate the usefulness of the PaO2/FIO2 ratio for predicting mortality in patients after cardiac surgery in order to provide a potential tool and value for immediate postoperative assessment. We also aimed to test the value of the PaO2/FIO2 ratio to assess respiratory complications.

## Methods

A prospective single-center observational study was conducted in a 10-bed cardiac surgery ICU at a 900-bed referral university hospital. The data were collected between January 2004 and January 2009. During the study period 2725 consecutive patients underwent various types of cardiac surgery. PaO2/FIO2 ratios after ICU admission were obtained for 2701 patients and these data were used for the subsequent analysis. Heart-transplant patients (*n* = 98) were not included, due to their particular physiopathology.

The local clinical research ethics committee (Comité d’Ètica i Assajos Clínics de Hospital Universitari de Bellvitge) approved the study protocol. Informed consent was waived because of the observational nature of the study and because all procedures were routine.

The PaO2/FiO2 ratio was measured at ICU admission, and after 3 h, 6 h, 12 h and 24 h, together with clinical data and outcomes. Data on and during ICU admission were extracted from the medical registry of each patient in real time using a standardized questionnaire and stored in a database for analysis. The definitions used for this study were based on the Society of Thoracic Surgeons’ national cardiac surgery database definitions [15]. In all the patients admitted to our cardiac surgery ICU we recorded demographic data (including risk factors for cardiovascular disease), diagnostic category, preoperative conditions, type of surgery (valvular, coronary or both) and characteristics (cardiopulmonary bypass (CBP) and aortic clamping times), respiratory complications, length of ICU and hospital stay, and mortality in the ICU and hospital. Outcome scores were also calculated for each patient: cardiac surgery scores (Parsonnet and EuroSCORE) and ICU scores (APACHE II and III, and SAPS II).

Both the clinical and laboratory data obtained are based on the internal postoperative protocol in place when this study was performed. Arterial blood gas analyses were performed at our hospital’s local laboratory, which meets the International Organization for Standardization quality standards (ISO 9001:2000).

The operations were performed by the same group of cardiac surgeons. Cardiac procedures were performed in all patients using median sternotomy, standard CPB with moderate hypothermia (34°C) and antegrade cardioplegia. A mean aortic pressure of > 60 mmHg was maintained during surgery. Intraoperative ventilatory strategies were based on an individual approach according to the patient’s previous respiratory status. Volume-controlled ventilation with a tidal volume of around 8 mL · kg^-1^ and a minimum PEEP were used to provide adequate ventilation and oxygenation, to prevent atelectasis and to maintain inspiratory plateau pressure <30 cmH_2_O. Minimum FiO2 was used to guarantee adequate oxygenation, even in the presence of CPB. All ventilatory parameters were modified in accordance with intraoperative analyses. For revascularization the internal thoracic artery was used (or bilaterally if possible) and saphenous vein grafts. Bypass graft flow was assessed for each graft by Doppler transit time flowmetry. Protamine was administered to reverse heparin, in accordance to standard practice. For Coronary Artery Bypass Graft (CABG) surgery, aspirin was routinely administered within the first 6 h after surgery following the local protocol. In all patients, decisions regarding postoperative ICU management were made by the attending physician. Patients were treated according to hemodynamic parameters, urine output, metabolic markers of tissue perfusion, such as arterial lactate levels and venous oxygen saturation, and an individual mechanical ventilation approach was performed in accordance with respiratory status.

### Statistical analyses

Statistical analysis was conducted using PASW statistics 13.0 (SPSS Inc., Chicago, Illinois, USA). Differences regarding PaO2/FIO2 ratios between survivors and non-survivors were evaluated by means of repeated measures analysis of variance. Receiver operating characteristic (ROC) curve analyses were applied to evaluate the predictive power between different PaO2/FIO2 ratio values and considering the differences of the areas under the empirical ROC curves (AUC). In order to determine optimal cut-off values of the best predictive PaO2/FIO2 ratio value, optimum threshold estimation was applied.

The optimum threshold was estimated by means of an adequately weighed cost function, which was then minimized [16]. A confidence interval was also estimated for this threshold, such that patients with values below the lower confidence interval limit were predicted to be non-survivors, patients with values above the upper confidence interval limit were predicted to be survivors, and values between the two limits were considered as inconclusive. Prognostic indexes, such as sensitivity and specificity and the likelihood ratio for survival were also calculated.

We thus studied all the data based on groups generated from previous analyses. For comparisons between groups, post-hoc exploratory analysis comparing different baseline and clinical characteristics was performed. ANOVA (P shown in tables) with post-hoc Bonferroni correction (P shown in results) was applied in order to evaluate any differences between the three groups for the quantitative samples with a normal distribution (e.g., TnI, ICU stay in hours, etc.). For qualitative variables (e.g., mortality), the χ^2^-test test was used. These differences were confirmed by means of multivariate analysis after adjusting for preoperative and postoperative scores. The multivariate analysis was a proportional hazards Cox regression model to evaluate the effect of staging in the different three groups of PaO2/FIO2 ratio at 3 h and the differences between the subgroups. Variable selection was performed stepwise and a variable remained in the model if the *p*-value was <0.1. Model fit was assessed through checking residual normality, the existence and influence of outliers, and goodness-of-fit.

Finally, we evaluated the PaO2/FIO2 ratio as a mortality risk factor after cardiac surgery analysing differences between survivors and non-survivors. For this purpose we categorized PaO2/FIO2 ratios. For comparisons between groups the Mann–Whitney *U* test was used or, when appropriate, the two-sample *t*-test. The *χ*^*2*^-test was used to evaluate qualitative variables. Multivariate analysis was performed based on the previous methods. Survival analysis was carried out with the Kaplan-Meier estimator and confirmed by means of the proportional hazards Cox regression model. The normality of the quantitative samples was checked by means of the one-sample Kolmogorov-Smirnov test in all cases, if necessary. Data are expressed as mean ± standard deviation. A two-tailed *p* value < 0.05 was considered statistically significant.

## Results

**Figure 1:**
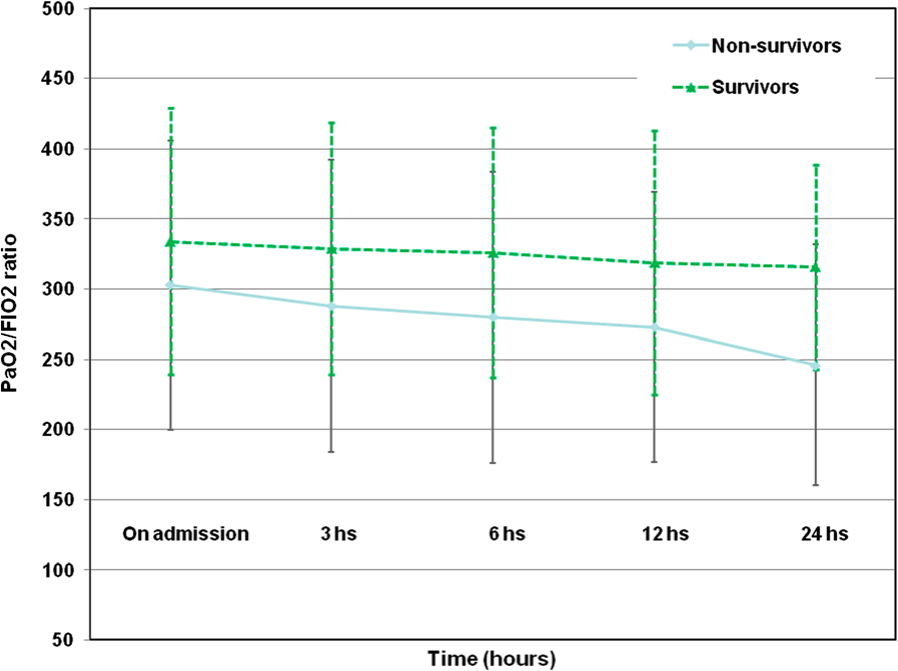
PaO2/FiO2 ratio levels curve of different measurements between survivors and non-survivors.

**Figure 2:**
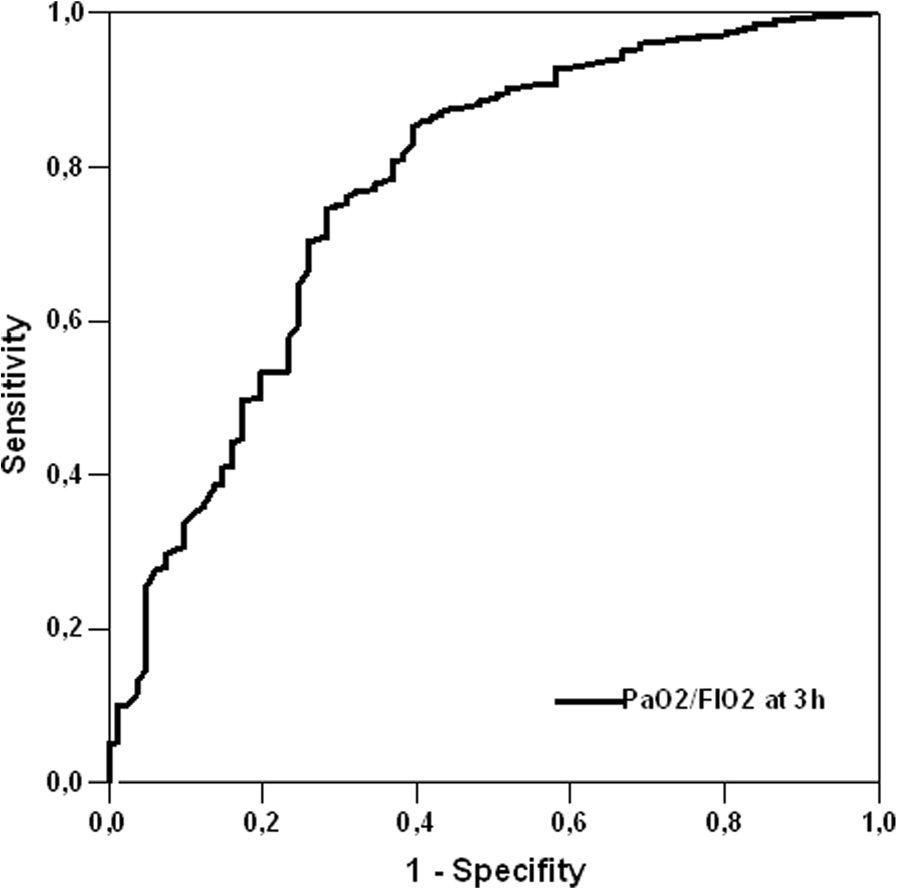
Receiver operating characteristic curve analysis for PaO2/FIO2 at 3 h.

PaO2/FIO2 ratios were higher in survivors than non-survivors (see Figure 1 and Table 1). ROC curve analysis showed that the PaO2/FIO2 ratio at 3 h after ICU admission was the best predictor of ICU mortality, and was also better than cardiac surgery scores. When comparing ROC curves of the PaO2/FIO2 ratio at 3 h with ICU scores, there were only slight differences, except in the case of APACHE II which was considerably higher (see Figure 2 and Table 2).

**Table 1:** PaO2/FiO2 ratio levels curve of different measurements between survivors and non-survivors

	**Survivors (n = 2569; 95.1%)**	**Non-survivors (n = 132; 4.9%)**	***P***
PaO2/FIO2 ratio on admission	334 ± 95	303 ± 103	***0.001***
PaO2/FIO2 ratio at 3 h	329 ± 90	288 ± 104	***<0.001***
PaO2/FIO2 ratio at 6 h	326 ± 89	280 ± 104	***<0.001***
PaO2/FIO2 ratio at 12 h	319 ± 94	273 ± 96	***0.008***
PaO2/FIO2 ratio at 24 h	316 ± 73	246 ± 86	***<0.001***

PaO2/FiO2 ratio: Arterial partial pressure of O2 and fraction of inspired oxygen ratio.

**Table 2:** Comparison of area under curve (AUC) for the different PaO2/FIO2 ratios and scores

	**AUC ± SD% (95% CI)**	**Cut-off levels**	**Sensitivity**	**Specificity**	***P*** **value**
PaO2/FIO2 ratio on admission	58.8 ± 8.8 (54.6 - 66.9)	314	63%	58%	***0.001***
PaO2/FIO2 ratio at 3 h	77.2 ± 2.9 (71.2 - 82.8)	255	81%	69%	***<0.001***
PaO2/FIO2 ratio at 6 h	67.3 ± 3.2 (60.9 - 73.6)	301	61%	68%	***<0.001***
PaO2/FIO2 ratio at 12 h	71.1 ± 3.2 (64.9 - 77.6)	265	70%	64%	***<0.001***
PaO2/FIO2 ratio at 24 h	63.4 ± 3.3 (57.0 - 69.8)	300	62%	57%	***<0.001***
EuroSCORE	71.1 ± 4.4 (62.5 - 79.7)	6.5	69.4%	56.3%	***<0.001***
Parsonnet	68.7 ± 5.1 (58.8 - 78.7)	12.5	63.9%	66.4%	***<0.001***
SAPS II	78.5 ± 4 (70.6 - 86.4)	27.5	80.6%	70%	***<0.001***
APACHE II	82 ± 3.9 (74.4 - 89.6)	13.5	72.2%	63.3%	***<0.001***
APACHE III	79.6 ± 4.5 (70.8 - 88.4)	51.5	75%	62.1%	***<0.001***

PaO2/FiO2 ratio: Arterial partial pressure of O2 and fraction of inspired oxygen ratio. EuroSCORE: European system for cardiac operative risk evaluation. SAPS: Simplified Acute Physiology Score. APACHE: Acute Physiology and Chronic Health Evaluation.

Results are expressed as mean ± standard deviation or percentage.

Comparison of area under curve (AUC) for the different PaO2/FIO2 ratios and scores.

The optimum threshold estimation explored the value of the PaO2/FIO2 ratio at 3 h after admission as a predictor of hospital mortality, giving an optimal cut-off of 222 (95% Confidence interval (CI):202–242). On the basis of the confidence interval limits, patients were classified into three groups: patients with a PaO2/FIO2 ratio >241, corresponding to a high expectation of survival (Group 1); patients with a PaO2/FIO2 ratio between 202 and 241, corresponding to an inconclusive outcome (Group 2); and patients with a PaO2/FIO2 ratio <202, corresponding to low expectation of survival (Group 3). The results showed 2195 patients with a PaO2/FIO2 ratio >241, 257 with a ratio between 202 and 241, and 249 with a ratio <202. The accuracy indexes of sensitivity and specificity were 82% and 21% respectively, and the predictive value for in hospital survival was 96%. The likelihood ratio for survival was 1.23 (95% CI:1.09-1.39) and for the outcome death was 0.41 (95% CI:0.29-0.58). These results indicate that survival is more likely in a patient with a PaO2/FIO2 ratio higher than 242, and death is more likely in a patient with a ratio lower than 202.

Baseline demographic and clinical characteristics of different groups (preoperative, intraoperative, and postoperative) are shown in Table 3. Regarding the preoperative data, Group 2 had a higher percentage of males (Bonferroni post-hoc: *p* < 0.001). The incidence of chronic obstructive bronchopulmonary disease (COPD) was higher in Group 3 (21.7%) than in either Group 2 (17.5%) or Group 1 (9.9%) (*p* < 0.001). There was a higher percentage of smokers in Group 2 (28.4%) than in Group 3 (26.5%) and Group 1 (19.8%) (*p* < 0.001). Group 3 patients tended to have higher body mass indices than patients in both groups 2 and 3, although the difference was not significant. Patients in Group 3 had higher preoperative creatinine levels and more chronic renal failure (CRF) than those in the other two groups (*p* = 0.001). As well, TnI levels were higher in Group 3 than among patients in the other two groups (*p* < 0.001). Scores on all the prognostic systems (Parsonnet and EuroSCORE, SAPS II, APACHE II, APACHE III) were likewise higher in Group 3.

**Table 3:** Baseline demographic and clinical characteristics (preoperative, intraoperative, and postoperative) of patients

	**PaO**_**2**_**/FIO**_**2**_	**PaO**_**2**_**/FIO**_**2**_	**PaO**_**2**_**/FIO**_**2**_	***p*** **value**
	**ratio > 241**	**ratio 202-241**	**ratio < 202**	
	**(*n*** **= 2195)**	**(*n*** **= 257)**	**(*n*** **= 249)**	
Preoperative data				
Age	65.3 (11.1)	66.1 (9.6)	66.0 (10.2)	*0.392*
BMI (kg∙m^-2^)	27.9 (8.5)	28.8 (4.2)	29.7 (17.5)	*0.09*
Gender *(% male)*	60.1	73.5	72.7	***< 0.001***
HBP (%)	61.8	67.7	66.7	***<0.001***
IDDM (%)	8.2	9.3	8.0	*0.867*
NIDDM (%)	17.5	17.9	18.1	*0.748*
DLP (%)	51.2	56.4	47.8	*0.354*
Smokers (%)	19.8	28.4	26.5	***<0.001***
Vasculopathy (%)	8.4	8.9	9.6	*0.947*
COPD (%)	9.9	17.5	21.7	***<0.001***
CRF (%)	4.1	5.1	10.4	***<0.001***
Ejection fraction (%)	60.7 (11.5)	58.7 (13.0)	60.0 (11.9)	*0.06*
Preoperative haematocrit (%)	39.5 (4.9)	40.5 (5.0)	38.8 (6.0)	***<0.001***
Preoperative platelets (x10^6^/L)	216423 (67126)	213470 (60195)	219409 (79665)	*0.615*
Preoperative creatinine *(μmol/l)*	91.8 (48.5)	94.6 (37.1)	107.9 (80.8)	***<0.001***
Parsonnet	10.8 (6.4)	11.5 (8.0)	13.8 (9.9)	***<0.001***
EuroSCORE	5.2 (2.8)	5.5 (3.2)	6.2 (3.6)	***0.006***
Surgical group				
CABG (%)	28.6	35.8	31.1	*0.145*
Valve (%)	62.7	55.3	60.7	*0.145*
CABG + Valve (%)	8.7	8.9	8.2	*0.145*
CPB time *(min)*	109.9 (36.4)	115.1 (37.6)	118.6 (40.7)	***<0.001***
Cross-clamping time *(min)*	71.9 (26.6)	73.2 (24.6)	76.0 (29.7)	*0.07*
Main Postoperative data				
SAPS II	22.8 (8.9)	26.4 (9.0)	29.3 (10.4)	***<0.001***
APACHE II	11.7 (4.2)	13.0 (4.8)	14.7 (5.4)	***<0.001***
APACHE III	47.6 (16.2)	52.8 (18.5)	60.8 (20.7)	***<0.001***
Blood lactate at ICU admission *(mmol/L)*	2.3 (5.6)	2.3 (1.2)	2.4 (1.4)	*0.960*
TnI at ICU admission *(μg/L)*	6.5 (7.0)	7.0 (11.8)	9.4 (25.5)	***<0.001***
Need for blood products (units)	1.8 (2.5)	1.4 (2.8)	1.9 (3.1)	*0.17*
ICU stay (days)	6.4 (9.2)	8.48 (11.6)	12.5 (15.1)	***<0.001***
Hospital stay (days)	23.6 (19.5)	24.7 (18.0)	31.7 (23.0)	***<0.001***
In-ICU exitus (%)	2.6	4.7	8.4	***< 0.001***
In-hospital exitus (%)	4	6.2	11.2	***<0.001***

*PaO2/FiO2* Arterial partial pressure of O2 and fraction of inspired oxygen ratio, *HBP* High blood pressure, *IDDM* Insulin-dependent diabetes mellitus, *NIDDM*, Non-insulin-dependent diabetes mellitus, *DLP* Dyslipidaemia, *COPD,* Chronic obstructive pulmonary disease, *CRF* Chronic renal failure, *BMI* Body Mass Index, *EuroSCORE* European system for cardiac operative risk evaluation, *SAPS* Simplified Acute Physiology Score, *APACHE* Acute Physiology and Chronic Health Evaluation, *CPB* Cardiopulmonary bypass, *CABG* Coronary artery bypass graft, *TnI* Troponin I, Data expressed as mean (SD).

**Figure 3:**
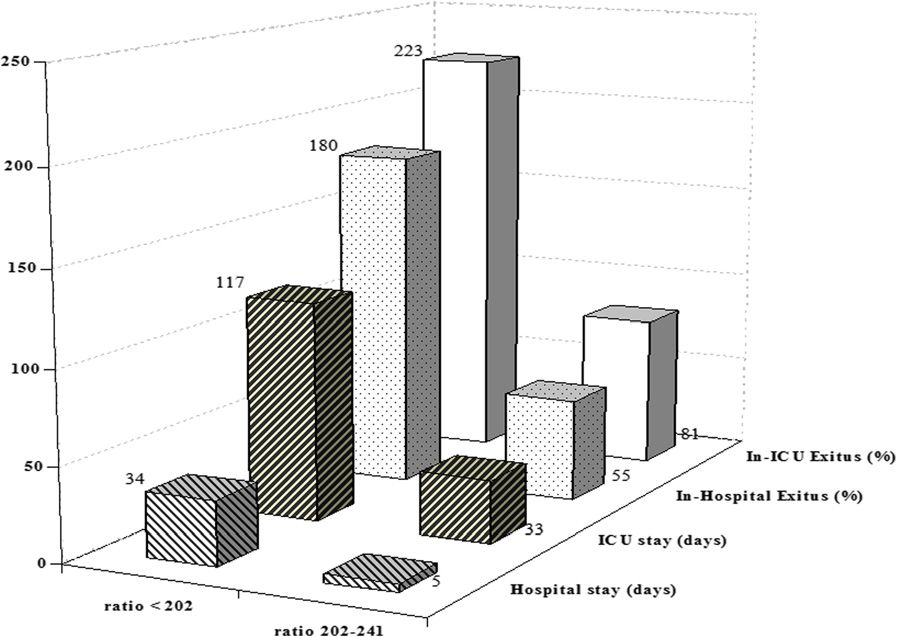
**Percentage of exitus and stay in PaO**_**2**_**/FiO**_**2**_**ratio <202 group and PaO**_**2**_**/FiO**_**2**_**ratio 202–241 group, compared to PaO**_**2**_**/FiO**_**2**_**ratio >241 group.**

Regarding outcomes, the mean ICU stay in Group 3 was 4.1 days longer than in Group 2 (*p* = 0.002) and 6.1 days longer than in Group 1 (*p* < 0.001). Similarly, the hospital stay in Group 3 was 7 days longer by than in Group 2 and 8.1 days longer than in Group 1 (both *p* < 0.001). Group 3 showed significantly greater in-ICU mortality (χ^2^ = 25.2, *p* < 0.001) and in-hospital mortality (χ^2^ = 26.2, *p* < 0.001) compared with the other two groups. The differences between groups in terms of mortality and length of stay are represented in Figure 3. In-ICU mortality was 320% higher in patients with a PaO2/FIO2 ratio <202 than in patients with a PaO2/FIO2 ratio >241. The same pattern was observed for in-hospital mortality, which was 280% higher in patients with a PaO2/FIO2 ratio <202 compared with those with a ratio > 241. In addition, both ICU and hospital stay were longer in patients with a ratio <202 than in those with a ratio > 241: longer by 6 days for ICU stay and by 8 days for hospital stay. Finally, regarding respiratory complications, Group 2 and 3 also showed higher rates (see Table 4) with the exception of diaphragmatic paresis and hemothorax.

**Table 4:** Respiratory data and complications during ICU admission after cardiac surgery

	**PaO**_**2**_**/FIO**_**2**_	**PaO**_**2**_**/FIO**_**2**_	**PaO**_**2**_**/FIO**_**2**_	***p*** **value**
	**ratio > 241**	**ratio 202-241**	**ratio < 202**	
	**(*n*** **= 2195)**	**(*n*** **= 257)**	**(*n*** **= 249)**	
Mechanical ventilation (hours)	35 (101)	59 (136)	125 (200)	***<0.001***
Hours until tracheal extubation	27.5 (74.1)	42.8 (86.9)	70.5 (122.9)	***<0.001***
Maximum PEEP needs	3.4 (1.4)	4.4 (4.7)	5.7 (2.5)	***<0.001***
Pneumonia	0.2	1.2	0.4	***0.046***
Hemothorax	0.2	0	0.4	*0.57*
Pneumothorax	2.2	3.9	6	***0.001***
Acute Pulmonary Edema	5.4	6.6	15.7	***<0.001***
Tracheostomy	4.1	6.2	17.7	***<0.001***
Tracheal reintubation	2.6	2.3	5.2	***0.003***
Diaphragmatic paresis	0.5	0.8	1.2	*0.43*
Pleural effusion	6.6	10.9	16.5	***<0.001***

PEEP: positive end expiratory pressure. Data expressed as mean (SD).

Multivariate analysis (see Table 5) confirmed higher tracheal reintubation rates in Groups 2 and 3 than in Group 1; higher Acute Pulmonary Edema in Group 3 wthan Group 1; and the highest need for tracheostomy in Group 3. It also confirmed that Group 3 had the highest in-ICU mortality and ICU length of stay, and higher CRF and COPD rates than in group 1.

**Table 5:** **Differences between subgroups according PaO**_**2**_**/FIO**_**2**_**ratio at 3 h after surgery in a multivariate analysis**

	**Hazard ratio (95% CI)**	***P value***
**PaO**_**2**_**/FIO**_**2**_**ratio > 241** subgroup vs. **PaO**_**2**_**/FIO**_**2**_**ratio 202–241** subgroup		
Preoperative haematocrit (%)	1.080 (1.054 - 1.108)	***<0.001***
Tracheal reintubation	5.338 (1.938 – 14.707)	***0.001***
ICU stay (days)	1.972 (1.959 - 1.985)	***<0.001***
In-ICU exitus	1.348 (1.185 - 1.654)	***0.001***
**PaO**_**2**_**/FIO**_**2**_**ratio > 241** subgroup vs. **PaO**_**2**_**/FIO**_**2**_**ratio < 202** subgroup		
Acute pulmonary edema	1.636 (1.418 - 1.968)	***0.035***
Tracheal reintubation	2.649 (1.147 - 6.116)	***0.022***
Need of tracheostomy	2.121 (1.015 - 4.432)	***0.046***
ICU stay (days)	1.961 (1.951 - 1.971)	***<0.001***
In-ICU exitus	1.332 (1.206 - 1.536)	***<0.001***
**PaO**_**2**_**/FIO**_**2**_**ratio 202–241** subgroup vs. **PaO**_**2**_**/FIO**_**2**_**ratio < 202** subgroup		
Chronic obstructive pulmonary disease	1.031 (1.006 - 1.056)	***0.015***
Chronic renal failure	1.847 (1.371 - 2.207)	***0.005***
Need of tracheostomy	2.371 (1.301 - 4.322)	***0.005***
ICU stay (days)	1.489 (1.319 - 1.602)	***<0.001***
In-ICU exitus	1.190 (1.078 - 1.459)	***<0.001***

**Figure 4:**
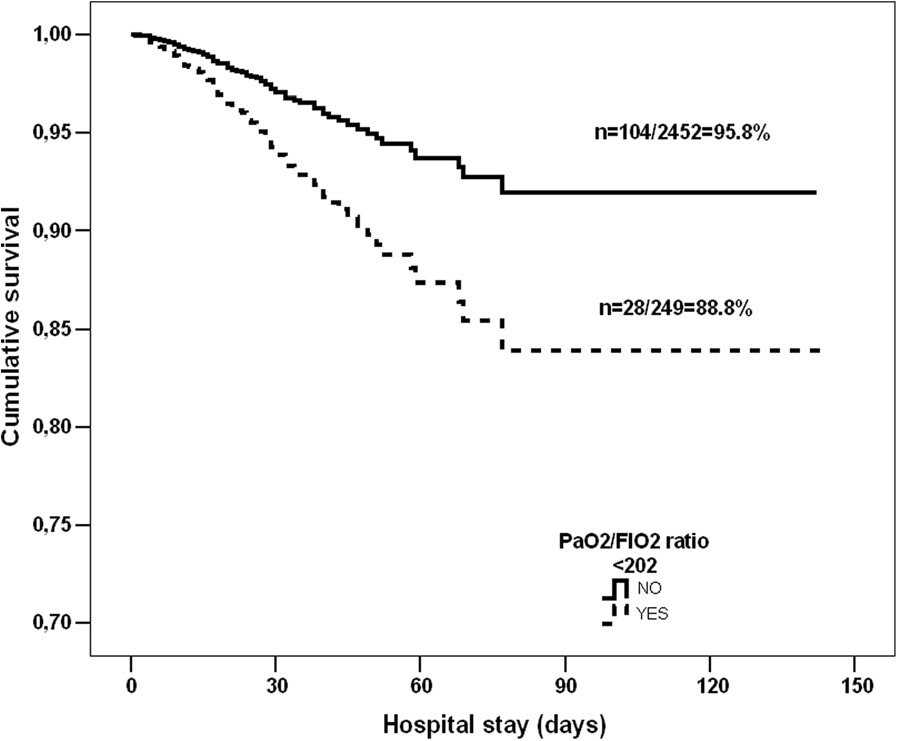
Kaplan-Meier survival curves for PaO2/FIO2 ratio <202 at 3 h subgroup.

When we categorized the presence of a PaO2/FIO2 ratio <202 at 3 h after admission, we confirmed its value as a predictor of in-ICU mortality (OR: 1.364; 95% CI: 1.212-1.625, *p <* 0.001). Kaplan-Meier plots, shown in Figure 4, illustrated that patients with PaO2/FIO2 ratio <202 at 3 h after admission had the poorest long-term survival over the hospital stay (Log rank test: *p* = 0.002), which was confirmed by means of multivariate analysis (Adjusted Hazard ratio: 1.48; 95% CI:1.293 – 1.786; *p* = 0.004).

## Discussion

Using a new method, the optimum threshold estimation criterion, this study highlights the value of the PaO2/FIO2 ratio as a prognostic indicator after cardiac surgery. The main findings of our study are the association between higher in-ICU mortality and length of stay with lower PaO2/FIO2 ratios.

PaO2/FIO2 values were lower in non-survivors than in survivors. Furthermore, the ratio has been reported to be a predictor of mortality in critically ill patients, particularly in patients with acute lung injury and in ARDS [5-7]. Low values of the PaO2/FIO2 ratio may be due to pathological conditions, primarily those of a respiratory nature (atelectasis, ARDS, acute pulmonary edema, pneumonia, etc.), as well as to alterations in hemodynamic status (cardiogenic shock, septic shock, etc.), or even both.

Immediately after cardiac surgery it is easy for physicians to detect a low PaO2/FIO2 ratio which can be swiftly corrected using general ICU procedures, such as appropriate analgesia, correcting tracheal tube placement if needed, or increasing PEEP to correct intraoperative atelectasis [8]. The same applies to hemodynamic disturbances. These potentially reversible causes may confound the immediate evaluation of the patient’s status performed on admission. Hence, the PaO2/FIO2 ratio 3 h after ICU admission is the most useful for prognosis purposes.

The so-called “fast-track”, modifying anesthetic techniques and postoperative sedation protocols, may allow for early tracheal extubation and, therefore, early ICU discharge without a significant increase in morbidity or mortality when correctly applied [17,18]. However, readmission after this procedure, though infrequent, produces a catastrophic increase in both mortality and the length of ICU stay [19,20]. In our view, the use of the PaO2/FiO2 ratio may help to minimize these types of events if specific strategies for weaning from mechanical ventilation are developed for patients at risk.

Several scoring systems are used to predict outcome in patients undergoing cardiac surgery, notably the Parsonnet score and the EuroSCORE [13,14], while for patients admitted to ICUs the SAPS II, APACHE II, and APACHE III are used [9,21]. Although the Parsonnet score and EuroSCORE are useful for determining the outcome of patients undergoing cardiac surgery they are both based on preoperative variables and so do not evaluate intraoperative or postoperative conditions and/or complications. SAPS II, APACHE II, and APACHE III are useful in determining the outcome of ICU patients, but they are only available 24 h after ICU admission and require the determination of several physiopathological variables and analytical parameters, including the PaO2/FIO2 ratio. Therefore, in the immediate postoperative period physicians lack a prognostic tool other than their own clinical judgment. In addition, cardiac surgery patients constitute a specific population of ICU patients with low mortality and short length of stay; it is therefore crucial to detect the ones who are at risk of postoperative complications and/or death, especially considering the increase in aging and comorbidities in previous years [22].

The presence of preoperative organ dysfunction adversely affects outcome after cardiac surgery [23]. Minimal changes in preoperative kidney function are associated with a substantial increase in the risk of mortality and morbidity following cardiac surgery, even when increases in serum creatinine levels are minimal [24]. In addition, during Cardiopulmonary Bypass (CPB) the kidneys may suffer from an imbalance between oxygen supply and oxygen needs, resulting in inadequate oxygen delivery that can be associated with worsening of renal function [25]. In addition, the presence of COPD entails a variable degree of airway inflammation which may be aggravated by CPB [26]. Both COPD and CRF show a consistent trend of increasing frequency of postoperative complications with advanced disease [23]; the higher incidences of both chronic diseases in the PaO2/FIO2 group with the worst survival may be related to this.

Respiratory complications are frequent reasons for ICU readmission and increase length of stay and mortality [27]. In consequence, our findings for respiratory complications are not surprising, especially if we consider PaO2/FIO2 ratio as a reflection of respiratory status. Tracheal reintubation worsens outcomes, increasing both complications and mortality [28]. The higher risk of tracheal reintubation in the groups with lower PaO2/FIO2 ratios may promote the use of the ratio as an assessment tool in tracheal extubation and/or weaning strategies. Early tracheostomy in patients who require prolonged mechanical ventilation after cardiac surgery is associated with decreased length of stay, morbidity, and mortality [27,28]. We suggest this strategy in the case of a persistently low PaO2/FIO2 ratio after an individual approach.

It was not the purpose of our study to differentiate between causative conditions of low PaO2/FIO2 ratio. Our results do not specifically address the cause of a low PaO2/FIO2 ratio in patients after cardiac surgery (which may represent a limitation of the study), but serve to evaluate the outcome in this scenario. Our study has other limitations as well. The most important is that it was a single-centre, observational study. However, it was conducted at a large tertiary referral hospital with a high level of complexity which performs all types of cardiac surgery and has a referral population of almost 2 million. Among the strengths of this study are its large sample size (the largest to date in studies addressing the postoperative PaO2/FIO2 ratio) and the prospective design. Our results may be clinically relevant, since a simple determination of PaO2/FIO2 ratio may provide very important information for determining outcome, both in terms of mortality and length of stay, in the immediate postoperative care of cardiac surgery patients. In addition, this strategy may be applicable in other post-cardiac surgery ICU settings because all the procedures and measurements are routine in clinical practice and easy to obtain.

## Conclusions

In summary, the PaO2/FIO2 ratio may be useful for identifying cardiac surgery patients at risk in the immediate postoperative period. PaO2/FIO2 ratios are lower in patients at risk, and the values at 3 h after admission are the most useful in terms of predicting in-ICU outcome. Respiratory complications are more frequent with PaO2/FIO2 ratios lower than 241. A simple determination of PaO2/FIO2 ratio at 3 h may provide important information about patient status. Physicians should be alert to the presence of low values, especially PaO2/FIO2 ratios at 3 h below 202.

### Key messages

• The PaO2/FIO2 ratio may be useful to identify at risk cardiac surgery patients in the immediate postoperative period.

• The PaO2/FIO2 ratio at 3 h after ICU admission has the highest prediction power in terms of outcome.

• Respiratory complications are more frequent when the PaO2/FIO2 ratio at 3 h is lower than 241 and poor outcome when the ratio below 202.

## Abbreviations

PaO2/FiO2 ratio: Arterial partial pressure of O2 and fraction of inspired oxygen ratio; ARDS: Respiratory distress syndrome in adults; ICU: Intensive care unit; EuroSCORE: European system for cardiac operative risk evaluation; APACHE: Acute physiology and chronic health evaluation; SAPS: Simplified acute physiology score; CPB: Cardiopulmonary bypass; CABG: Coronary artery bypass graft; COPD: Chronic obstructive bronchopulmonary disease.

## Competing interests

The authors declare that they have no competing interest.

## Authors’ contributions

FE was involved in the conception and design of the research, as well as performed statistical analysis and wrote the paper. JCLD performed partial statistical analysis and wrote the paper. CJ performed statistical analysis and interpretation of data. KS supervised and performed statistical analysis. DRC and HT were involved in the coordination and the acquisition of data. MLC and EF contributed to the design of the research and acquisition of data. JLV was involved in the conception, design of the research and interpretation of data. RM and ADP were involved in the design of the research and supervised the writing of the present manuscript. All authors read and approved the final version of this manuscript.

## Pre-publication history

The pre-publication history for this paper can be accessed here:

http://www.biomedcentral.com/1471-2253/14/83/prepub
